# MultiXC-QM9: Large dataset of molecular and reaction energies from multi-level quantum chemical methods

**DOI:** 10.1038/s41597-023-02690-2

**Published:** 2023-11-08

**Authors:** Surajit Nandi, Tejs Vegge, Arghya Bhowmik

**Affiliations:** Department of Energy Conversion and Storage, Technical University of Denmark, Anker Engelunds Vej 301, 2800 Kongens Lyngby, Copenhagen, Denmark

**Keywords:** Cheminformatics, Thermodynamics

## Abstract

Well curated extensive datasets have helped spur intense molecular machine learning (ML) method development activities over the last few years, encouraging nonchemists to be part of the effort as well. QM9 dataset is one of the benchmark databases for small molecules with molecular energies based on B3LYP functional. G4MP2 based energies of these molecules were published later. To enable a wide variety of ML tasks like transfer learning, delta learning, multitask learning, etc. with QM9 molecules, in this article, we introduce a new dataset with QM9 molecule energies estimated with 76 different DFT functionals and three different basis sets (228 energy numbers for each molecule). We additionally enumerated all possible A ↔ B monomolecular interconversions within the QM9 dataset and provided the reaction energies based on these 76 functionals, and basis sets. Lastly, we also provide the bond changes for all the 162 million reactions with the dataset to enable structure- and bond-based reaction energy prediction tools based on ML.

## Background & Summary

The application of machine learning (ML) to predict molecular properties is well established now^1^. The effectiveness and proliferation of molecular ML models rest on large high-quality data sets. Datasets that are easy to access and machine actionable^2–4^ have enabled the broader community of machine learning researchers to participate in the building of novel molecular ML models^5–7^. The construction of small molecule properties databases that can be used to prototype and benchmark new ML architectures has been the basis of many ground-breaking machine learning developments in the context of predicting molecular properties^8–10^. Among many such datasets, QM9 dataset^11^ has been the gold standard for testing the latest models. In the original QM9 dataset the molecular electronic energies were reported with calculations based on density functional theory at the B3LYP/6–31 G (2df, p)^12–15^ level of theory. Recently, those were estimated with high accuracy composite quantum chemistry method (G4MP2)^16–18^. The availability of molecular energies at multiple levels of theory allows interesting ML tasks to be explored, such as the delta learning approach^19^, transfer learning^20^, and multi-task learning^21^.

Beyond the molecule property prediction task, ML can have a tremendous impact on chemical sciences by accelerating the prediction of reaction networks^22^. Building a reaction network requires very fast energy prediction as a large number of energy computation is necessary and thus DFT can be computationally too expensive for exploring large reaction networks. The use of low-level DFT method can reduce computational cost, but the low fidelity of predicted energies could lead to an erroneous analysis of the reaction network. Typically, semiempirical level theories, such as XTB are generally used, but those also have large errors. One way to mitigate this problem is to use energy-correcting methods to achieve higher accuracy via correcting energies from lower-level methods. Building ML-based delta correction models^23^ or simple statistical correction schemes^24^ that are widely applicable requires large datasets or reaction energies from both cheap/low-fidelity and expensive/high-fidelity methods.

With an aim of providing large datasets with energy targets from a wide variety of methods to help build both these novel molecular ML tasks and ML for reaction networks, we provide multilevel energies of the QM9 molecules and reactions derived from there. Molecular quantum chemistry community utilizes a wide variety of exchange correlation functional^25^ and thus any new ML method needs to be tested for predictions across many different XC to prove generalization and universality. Therefore, providing data for QM9 molecules with a number of DFT functionals and basis sets will provide new challenges to the ML community to build robust ML methods that can be applied to a variety of methods. Furthermore, we provide the energies with the GFN2-XTB method for the QM9 molecules and the reactions, keeping in mind that reaction network exploration methods often use this semi-empirical method.

## Methods

### Dataset generation

**Fig. 1:**
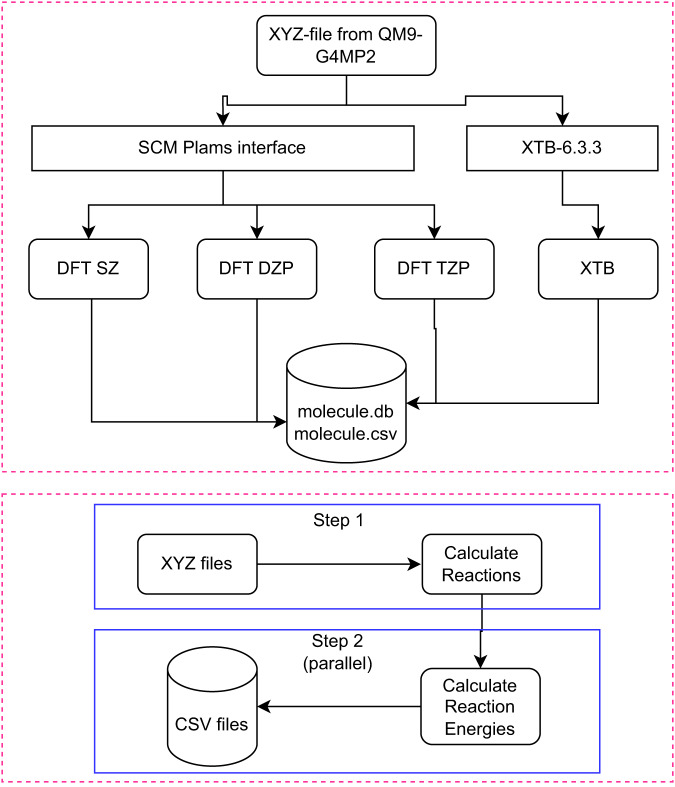
Semi-automated workflow diagram of the database preparation.

A semi-automated flowchart of data set generation is shown in Fig. 1. The entire process can be divided into approximately three steps. First, energy calculations were performed on the molecules using ADF(SCM) and XTB. Then, the energies were saved in the CSV and SQLite format using Python scripts. Atomistic simulation environment (ASE) was used to process the data and for energy calculation.

In the second step, all possible isodesmic reactions among the QM9 molecules were calculated. Two molecules are considered convertible if their chemical formula is the same. This means that the reactions in the dataset are of type A ↔ B. Once all the indices for the reactions are collected and saved in a csv file (“reactions.csv”), we computed the reaction energies and saved the results in multiple CSV files. Therefore, the creation of the datasets requires two steps: one, to compute the indices of the reactants and products, and then, to compute the reaction energies.

The xyz geometries of the QM9 molecules were extracted from the logfiles available in the Figshare repository^26^. We used those geometries to calculate the energies using the post-SCF method as implemented in the SCM software package. For all the post-SCF functionals, the GGA level energy was computed using the PBE method. We run three separate calculations with the SZ, DZP, and TZP basis set for the 133 K molecules. We also computed the single-point energy of the same geometries using the GFN2-XTB semi-empirical method as implemented in the xtb package. The SCF convergence criteria for the DFT calculation was the default one of ADF (1e-6 Hartree), and for the XTB calculation, the SCF convergence criteria were set to the default value of 1e-6 Hartree.

## Data Records

There are two types of data in the dataset - one contains information on molecules and the other contains information on reactions. The information of the molecules is stored in CSV and SQLite formats, while the information on the reactions is provided only in CSV format. The molecules are same as QM9 except that all the molecules with charge separation are excluded from the dataset. The energies are calculated as single point energies of the B3LYP/6–31 G(2df,p) optimized geometries from a previous dataset of the same molecules^16^. The geometries taken for the single point energy calculations were originally optimized at the B3LYP/6–31 G(2df,p) level^11^. It should be noted here that since the theoretical level at which the minima were computed is different from the one used in this article, the energies reported here may not correspond to the minimum energy geometries at the corresponding DFT level.

**Fig. 2:**
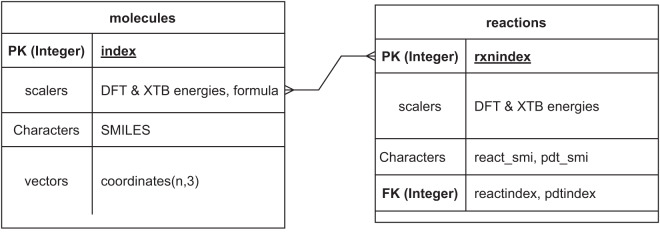
Entity relationship diagram of the molecule and reaction databases. The “index” and “rxnindex” represent the primary keys of the molecules and reactions data respectively. The “reactindex” and the “pdtindex” represents the foreign key and thus indicates the index of the molecules database.

The CSV format database contains energies from different DFT and semi-empirical methods, SMILES strings derived from the xyz files, and chemical formula of each of the species. Each molecules has a unique index number named “index”. The SQLite format molecular dataset contains xyz coordinates, energies, and other relevant properties which were derived automatically by ASE. The reaction database contains reaction energies, indices of the reactants and products, and SMILES of reactants and products. The reactant and product indices are the primary key (“index”) of the molecular database. Each reaction is identified with a key called “rxnindex”. The relationship between the molecular and reactions database is shown in Fig. 2. Each of the reactions are unique and even though the molecule to reaction cardinality is shown as many to many because, each reactions are related to two molecules (more than one).

The log files obtained from the energy calculations are publicly available from DTU Data^27^ (version 3 was accessed). All scripts to reproduce the reaction energies, databases, are available from GitHub under the MIT public license (https://github.com/chemsurajit/largeDFTdata). The log files are provided into four separate zip files; TZP.zip, DZP.zip, SZ.zip, XTB.zip. It should be noted that in the dataset by Kim *et al*.^16^, the author used the spin multiplicity of N as 2 when calculating the energy of a single atom. Since the spin multiplicity of the ground state of N is 4, we recalculated the atomization energies with the G4MP2 method after replacing the N.log file from the dataset with our own N.log file. We provide the N.log file for the calculation using Gaussian. The N.log file with the energy calculation using G4MP2 is provided in the dataset.

## Technical Validation

**Fig. 3:**
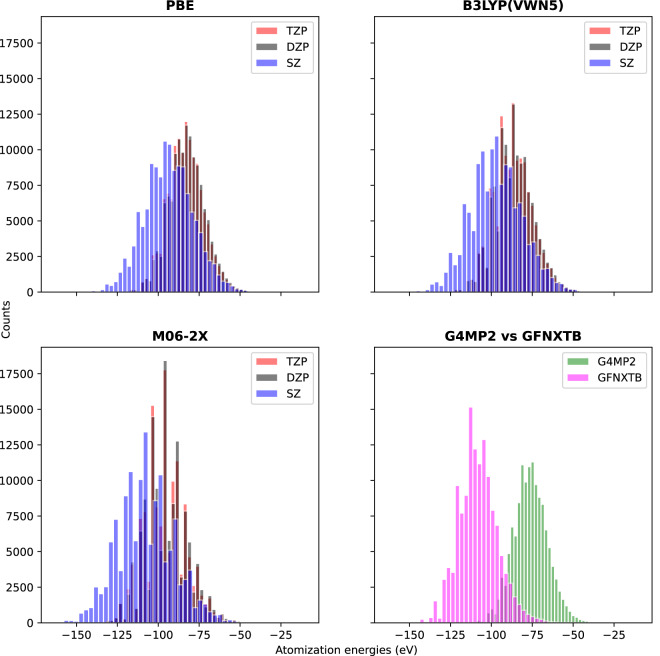
Atomization energy distribution of three density functionals: PBE (GGA type), B3LYP(VWN5) (hybrid functional) and M06-2X (highly parametrized meta-hybrid functional) in three different basis sets (SZ, DZP, and TZP). The last plot shows the atomization energy distribution difference between GFN2-xTB and G4MP2 method.

We presented the atomization energy distribution in two different ways. Due to the fundamental difference in calculating the atomization energies, we made a comparison first, among the basis sets of three different density functionals, second, between the G4MP2 and the GFN2-xTB methods. The atomization energy distribution plots are given in Fig. 3. In case of the DFTs, the atomization energies that correspond to the TZP and DZP basis sets are quite similar and the atomization energies from the SZ basis are relatively different. This is expected as the SZ basis is the minimalistic representation of the orbitals. It is also evident that the characteristic of the energy distribution varies among the functionals. Between the G4MP2 and GFN2-xTB energies, there are significant differences in the energy distribution, as expected.

**Fig. 4:**
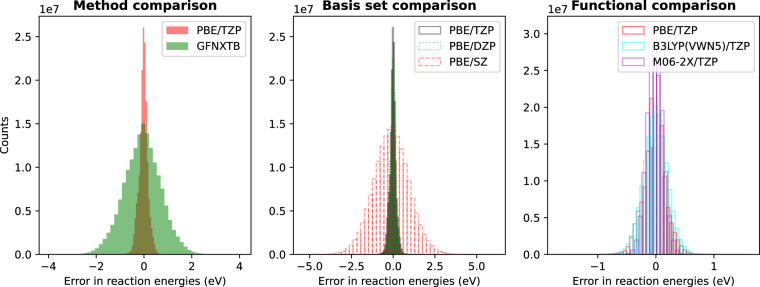
Distribution of the error in reaction energies (eV) with respect to the G4MP2 methods for different functionals.

Next, we show the distribution of the error in the reaction energies with respect to the G4MP2 reaction energies of different methods in Fig. 4. It is interesting, albeit expected, that the errors of the DFT functionals with the TZP and the DZP basis sets are similar and significantly small. The distribution of error in XTB reaction energies is similar to that of the DFT level with SZ basis set. Also, the error in energy distribution is mostly symmetric and of Gaussian type. This indicates that in any situation even though there can be large errors in atomization energies, the reaction energies mostly follow a normal distribution with the majority of reactions on a relatively small error scale. This type of behavior is known and expected since for a reaction cancelation of errors occurs^28^. Therefore, the errors in reaction energies become lower and sink to a scale that is much smaller than the atomization energies. It has to be noted here that the energies calculated by using the post-SCF method with the electron density calculated at the PBE level. Therefore, the accuracy of the energy values is expected to be less than the SCF calculations from each of the theoretical levels.

## Usage Notes

All the data are publicly available from DTU data^27^ (version 3 was accessed). Log files related to energy calculations are available from the data repository. Scripts for downloading and making the molecular and reaction database are available from the GitHub repository under the MIT license condition. The functional names are used as they are (even if they contain special characters) for the CSV file. For SQLite format, special characters in functional names are replaced by underscore(_). The changed functional names and the original name of the SCM output file are provided in Table S1 in the supporting information. The CSV file contains SMILES strings, index of the molecules, and the energy values. The SQLite format database contains the molecular coordinates, smile strings, and energy values. Since the SQLite format database is created with ASE, some other information of the molecules (e.g., chemical formula) is also available from this file.

The data on the reaction energies are provided as multiple CSV files. The CSV files contain reaction energies, reactant index (reactindex), product index (pdtindex), and a unique index (rxnindex) for each reaction. The index of the species is the same as the index in the database for molecules. Hence, if a model needs reactant and product coordinates, it can be read from the SQLite format database file of the molecule. Additionally, a README.md file is available in the GitHub repository explaining how to run the scripts.

### Supplementary information

Supporting Information Table

## Data Availability

The energy calculations with the 76 different post-SCF functionals were performed using the SCM software package^29^. The GFN2-xTB energies were computed using the XTB version 6.3.3 software package^30–32^. The G4MP2 energies were obtained from a previous paper by Kim *et al*.^16,26^. The workflow of the calculations and collection of data are build using the Python3.7.10 and BASH scripts. The atomistic simulation environment (ASE)^33^ was used to create the database file in SQLite3 format. The csv files were created using pandas. The plots were generated using the matplotlib library. All scripts are available on GitHub under the MIT license agreement (https://github.com/chemsurajit/largeDFTdata).
