# Supplementary material for: MultiXC-QM9: Large dataset of molecular and reaction energies from multi-level quantum chemical methods
Source: Sci Data. 2023 Nov 8;10:783. doi: 10.1038/s41597-023-02690-2 (PMC10632468; doi:10.1038/s41597-023-02690-2)
Supplement: Supplementary file 1 — Supporting Information Table [file 41597_2023_2690_MOESM1_ESM.pdf]

MultiXC-QM9: Large dataset of molecular and reaction energies from multi-level quantum chemical methods

Surajit Nandi   Tejs Vegge   Arghya Bhowmik

List of the functionals

Functional name	Details
KCIS-MODIFIED[1]	Modified version of KCIS functional
KCIS-ORIGINAL[1]	Krieger-Chen-Iafrate-Savin functional
PKZB[2]	Perdew, Kurth, Zupan and Blaha functional
VS98[3]	Voorhis-Scuseria functional
LDA(VWN)	Vosko-Wilk-Nusair[4] approximation for LDA
PW91[5–8]	The PW91 exchange and correlation.
BLYP[9–11]	Exchange Becke, Correlation Lee-Yang-Parr
BP[9,12]	The BP86 functional
PBE[13,14]	Perdew-Burke-Ernzerhof functional
RPBE[15]	Revised PBE by Hammer-Hansen-Norskov
REVPBE[16]	Revised PBE by Zhang-Wang
OLYP[17]	Exchange: OPTX from Henry-Cohen, correlation: PBE
FT97[18]	MetaGGA functional
BLAP3[18]	MetaGGA functional
HCTH/93[19–21]	Handy's functional refined with 93 systems
HCTH/120[19–21]	Handy's functional refined with 120 systems
HCTH/147[19–21]	Handy's functional refined with 147 systems
HCTH/407[19–21]	Handy's functional refined with 407 systems
BMTAU1[18]	MetaGGA functional
BOP[18]	MetaGGA functional

PKZBX-KCISCOR[1,2,22,23]	exchange: Perdew-Kurth-Zupan-Blahafunctional, Correlation: KCIS
VS98-X(XC)	Variation of the VS functional[3]
VS98-X-ONLY[3]	The exchange part from VS functional
BECKE00[24]	Becke's functional.
BECKE00X(XC)[24]	B Becke's functional
BECKE00-X-ONLY[24]	Becke's functional.
BECKE88X+BR89C	Becke's functional.
OLAP3	Meta-GGA functional
TPSS[25]	Tao-Perdew-Staroverov-Scuseria functional of meta-GGA type
MPBE[26]	Modified PBE functional by Adamo-Barone
OPBE[27]	GGA functional
OPERDEW[28,29]	OPERDEW
MPBEKCIS	A combination of MPBE and KCIS
MPW[30]	Modified PW by Adamo-Barone
TAU-HCTH[31]	τ-dependent functional of HCTH family
XLYP[32]	Functional XLYP
KT1[33]	Functional by Keal and Tozer
KT2[33]	Functional by Keal and Tozer
M06-L[34,35]	Minnesota functional by Yan-Truhlar
BLYP-D	BLYP functional[9,10] with Grimme's dispersion correction[36]
BP86-D	BP86 functional with Grimme's dispersion
PBE-D	PBE functional with Grimme's dispersion
TPSS-D	TPSS functional with Grimme's dispersion
B97-D	Semiempirical GGA functional[37]
REVTPSS[38]	The revised version of the TPSS functional by Perdew et. al.
PBESOL[39]	GGA functional by Perdew-Ruzsinszky-Csonka-Vydrov-Scuseria
RGE2	Regularized gradient expansion functional
SSB-D[40,41]	Dispersion corrected functional by Swart-Solà-Bickelhaupt
MVS[42]	Functional by Sun-Perdew-Ruzsinszky
MVSX	
T-MGGA[18]	
TPSSH[25,43]	TPSS functional with 10% HF exchange
B3LYP(VWN5)[44]	B3LYP by Stephens-Devlin-Chablowski-Frisch
O3LYP(VWN5)[45]	O3LYP functional

KMLYP(VWN5)[46,47]	
PBE0[48,49]	Hybrid form of PBE
B3LYP*(VWN5)[50]	Modified B3LYP functional with 15% HF exchange
BHANDH	Hybrid functional with 50% exact exchange, 50% LDA exchange, and 100% LYP correlation. Exact exchange is based on M. A. Watson et al.[47]
BHANDHLYP	Hybrid functional with 50% exact exchange, 50% Becke88 exchange, and LYP correlation. Exact exchange is based on M. A. Watson et al.[47]
B97[51]	Hybrid functional by Becke
B97-1	Hybrid functional
B97-2[52]	Hybrid functional
MPBE0KCIS[53]	
MPBE1KCIS[53]	
B1LYP(VWN5)[54]	ADF version of B1LYP by Adamo-Barone
B1PW91(VWN5)[54]	Functional by Adamo-Barone
MPW1PW[55]	Functional by Adamo-Barone
MPW1K[56]	Functional by Lynch-Fast-Harris-Truhlar
TAU-HCTH-HYBRID[57]	Hybrid functional of τ-dependent HCTH functional
X3LYP(VWN5)[58]	Functional by Xu-Goddard
OPBE0[59]	Hybrid form of OPBE
M05[60]	Minnesota M05 meta-hybrid functional
M05-2X[61]	Minnesota M05-2X meta-hybrid functional
M06[62]	Minnesota M06 meta-hybrid functional
M06-2X[63]	Minnesota M06-2X meta-hybrid functional
B3LYP-D	B3LYP with Grimme's dispersion[36] included.
GFNXTB	The semiempirical XTB2 method.

Table S1: List of Actual keys and the modified keys for the energy calculation methods. The actual keys are used in the CSV files. The modified keys are used in the SQLite3 database file. The basis set information is added at the end of the functional with underscore.

Naming of the Functional in the Database

We have changed the name of the functionals (in Table S1) if any characters other than words and numbers are present, we replaced those characters with

underscore ("_"). We used the regular expression library (*re*) in python. The basis set used (SZ, DZP or TZP) are appended at the end of the functional name with an underscore. Thus, a energy calculation method "B3LYP(VWN5)/SZ" becomes "B3LYP_VWN5__SZ

References

[1] Krieger, J. B., Chen, J., Iafrate, G. J. & Savin, A. *Construction of An Accurate Self-interaction-corrected Correlation Energy Functional Based on An Electron Gas with A Gap* (Springer US, Boston, MA, 1999).

[2] Perdew, J. P., Kurth, S., Zupan, A. c. v. & Blaha, P. Accurate density functional with correct formal properties: A step beyond the generalized gradient approximation. *Phys. Rev. Lett.* **82**, 2544–2547 (1999).

[3] Van Voorhis, T. & Scuseria, G. E. A novel form for the exchange-correlation energy functional. *J. Chem. Phys.* **109**, 400–410 (1998).

[4] Vosko, S. H., Wilk, L. & Nusair, M. Accurate spin-dependent electron liquid correlation energies for local spin density calculations: a critical analysis. *Can. J. Phys.* **58**, 1200–1211 (1980).

[5] Perdew, J. P. *et al.* Atoms, molecules, solids, and surfaces: Applications of the generalized gradient approximation for exchange and correlation. *Phys. Rev. B* **46**, 6671–6687 (1992).

[6] Perdew, J. P. *et al.* Erratum: Atoms, molecules, solids, and surfaces: Applications of the generalized gradient approximation for exchange and correlation. *Phys. Rev. B* **48**, 4978–4978 (1993).

[7] Dobson, J. F., Vignale, G. & Das, M. P. Electronic density functional theory : recent progress and new directions (1998). URL `https://api.semanticscholar.org/CorpusID:92696156`.

[8] Perdew, J. P., Burke, K. & Wang, Y. Generalized gradient approximation for the exchange-correlation hole of a many-electron system. *Phys. Rev. B* **54**, 16533–16539 (1996).

[9] Becke, A. D. Density-functional exchange-energy approximation with correct asymptotic behavior. *Phys. Rev. A* **38**, 3098–3100 (1988).

[10] Lee, C., Yang, W. & Parr, R. G. Development of the colle-salvetti correlation-energy formula into a functional of the electron density. *Phys. Rev. B* **37**, 785–789 (1988).

[11] Miehlich, B., Savin, A., Stoll, H. & Preuss, H. Results obtained with the correlation energy density functionals of becke and lee, yang and parr. *Chem. Phys. Lett.* **157**, 200–206 (1989).

[12] Perdew, J. P. Density-functional approximation for the correlation energy of the inhomogeneous electron gas. *Phys. Rev. B* **33**, 8822–8824 (1986).

[13] Perdew, J. P., Burke, K. & Ernzerhof, M. Generalized gradient approximation made simple. *Phys. Rev. Lett.* **77**, 3865–3868 (1996).

[14] Perdew, J. P., Burke, K. & Ernzerhof, M. Generalized gradient approximation made simple. *Phys. Rev. Lett.* **78**, 1396–1396 (1997).

[15] Hammer, B., Hansen, L. B. & Nørskov, J. K. Improved adsorption energetics within density-functional theory using revised perdew-burke-ernzerhof functionals. *Phys. Rev. B* **59**, 7413–7421 (1999).

[16] Zhang, Y. & Yang, W. Comment on "generalized gradient approximation made simple". *Phys. Rev. Lett.* **80**, 890–890 (1998).

[17] Handy, N. C. & Cohen, A. J. Left-right correlation energy. *Mol. Phys.* **99**, 403–412 (2001).

[18] de Silva, P. & Corminboeuf, C. Communication: A new class of non-empirical explicit density functionals on the third rung of Jacob's ladder. *J. Chem. Phys.* **143**, 111105 (2015).

[19] Hamprecht, F. A., Cohen, A. J., Tozer, D. J. & Handy, N. C. Development and assessment of new exchange-correlation functionals. *J. Chem. Phys.* **109**, 6264–6271 (1998).

[20] Boese, A. D., Doltsinis, N. L., Handy, N. C. & Sprik, M. New generalized gradient approximation functionals. *J. Chem. Phys.* **112**, 1670–1678 (2000).

[21] Boese, A. D. & Handy, N. C. A new parametrization of exchange–correlation generalized gradient approximation functionals. *J. Chem. Phys.* **114**, 5497–5503 (2001).

[22] Krieger, J. B., Chen, J. & Kurth, S. Construction and application of an accurate self-interaction-corrected correlation energy functional based on an electron gas with a gap. *AIP Conf. Proc.* **577**, 48–69 (2001).

[23] Toulouse, J., Savin, A. & Adamo, C. Validation and assessment of an accurate approach to the correlation problem in density functional theory: The Kriger–Chen–Iafrate–Savin model. *J. Chem. Phys.* **117**, 10465–10473 (2002).

[24] Becke, A. D. Simulation of delocalized exchange by local density functionals. *J. Chem. Phys.* **112**, 4020–4026 (2000).

[25] Tao, J., Perdew, J. P., Staroverov, V. N. & Scuseria, G. E. Climbing the density functional ladder: Nonempirical meta–generalized gradient approximation designed for molecules and solids. *Phys. Rev. Lett.* **91**, 146401 (2003).

[26] Adamo, C. & Barone, V. Physically motivated density functionals with improved performances: The modified Perdew–Burke–Ernzerhof model. *J. Chem. Phys.* **116**, 5933–5940 (2002).

[27] Swart, M., Ehlers, A. W. & Lammertsma, K. Performance of the opbe exchange-correlation functional. *Mol. Phys.* **102**, 2467–2474 (2004).

[28] HANDY, N. C. & COHEN, A. J. Left-right correlation energy. *Mol. Phys.* **99**, 403–412 (2001).

[29] Perdew, J. P. Density-functional approximation for the correlation energy of the inhomogeneous electron gas. *Phys. Rev. B* **33**, 8822–8824 (1986).

[30] Adamo, C. & Barone, V. Exchange functionals with improved long-range behavior and adiabatic connection methods without adjustable parameters: The mPW and mPW1PW models. *J. Chem. Phys.* **108**, 664–675 (1998).

[31] Boese, A. D. & Handy, N. C. New exchange-correlation density functionals: The role of the kinetic-energy density. *J. Chem. Phys.* **116**, 9559–9569 (2002).

[32] Xu, X. & Goddard, W. A. The x3lyp extended density functional for accurate descriptions of nonbond interactions, spin states, and thermochemical properties. *Proc. Natl. Acad. Sci. U. S. A.* **101**, 2673–2677 (2004).

[33] Keal, T. W. & Tozer, D. J. The exchange-correlation potential in Kohn–Sham nuclear magnetic resonance shielding calculations. *J. Chem. Phys.* **119**, 3015–3024 (2003).

[34] Zhao, Y. & Truhlar, D. G. A new local density functional for main-group thermochemistry, transition metal bonding, thermochemical kinetics, and noncovalent interactions. *J. Chem. Phys.* **125**, 194101 (2006).

[35] Zhao, Y. & Truhlar, D. G. The m06 suite of density functionals for main group thermochemistry, thermochemical kinetics, noncovalent interactions, excited states, and transition elements: two new functionals and systematic testing of four m06-class functionals and 12 other functionals. *Theor. Chem. Acc.* **120**, 215–241 (2008).

[36] Grimme, S. Accurate description of van der waals complexes by density functional theory including empirical corrections. *J. Comput. Chem.* **25**, 1463–1473 (2004).

[37] Grimme, S. Semiempirical gga-type density functional constructed with a long-range dispersion correction. *J. Comput. Chem.* **27**, 1787–1799 (2006).

[38] Perdew, J. P., Ruzsinszky, A., Csonka, G. I., Constantin, L. A. & Sun, J. Workhorse semilocal density functional for condensed matter physics and quantum chemistry. *Phys. Rev. Lett.* **103**, 026403 (2009).

[39] Perdew, J. P. *et al.* Restoring the density-gradient expansion for exchange in solids and surfaces. *Phys. Rev. Lett.* **100**, 136406 (2008).

[40] Swart, M., Solà, M. & Bickelhaupt, F. M. A new all-round density functional based on spin states and SN2 barriers. *J. Chem. Phys.* **131**, 094103 (2009).

[41] Swart, M., Solà, M. & Bickelhaupt, F. M. Switching between optx and pbe exchange functionals. *J. Comput. Methods Sci. Eng.* **9**, 69–77 (2009).

[42] Sun, J., Perdew, J. P. & Ruzsinszky, A. Semilocal density functional obeying a strongly tightened bound for exchange. *Proc. Natl. Acad. Sci. U. S. A.* **112**, 685–689 (2015).

[43] Staroverov, V. N., Scuseria, G. E., Tao, J. & Perdew, J. P. Comparative assessment of a new nonempirical density functional: Molecules and hydrogen-bonded complexes. *J. Chem. Phys.* **119**, 12129–12137 (2003).

[44] Stephens, P. J., Devlin, F. J., Chabalowski, C. F. & Frisch, M. J. Ab initio calculation of vibrational absorption and circular dichroism spectra using density functional force fields. *J. Phys. Chem.* **98**, 11623–11627 (1994).

[45] COHEN, A. J. & HANDY, N. C. Dynamic correlation. *Mol. Phys.* **99**, 607–615 (2001).

[46] Kang, J. K. & Musgrave, C. B. Prediction of transition state barriers and enthalpies of reaction by a new hybrid density-functional approximation. *J. Chem. Phys.* **115**, 11040–11051 (2001).

[47] Watson, M. A., Handy, N. C. & Cohen, A. J. Density functional calculations, using Slater basis sets, with exact exchange. *J. Chem. Phys.* **119**, 6475–6481 (2003).

[48] Grimme, S. Accurate description of van der waals complexes by density functional theory including empirical corrections. *J. Comput. Chem.* **25**, 1463–1473 (2004).

[49] Ernzerhof, M. & Scuseria, G. E. Assessment of the Perdew–Burke–Ernzerhof exchange-correlation functional. *J. Chem. Phys.* **110**, 5029–5036 (1999).

[50] Reiher, M., Salomon, O. & Artur Hess, B. Reparameterization of hybrid functionals based on energy differences of states of different multiplicity. *Theor. Chem. Acc.* **107**, 48–55 (2001).

[51] Becke, A. D. Density-functional thermochemistry. V. Systematic optimization of exchange-correlation functionals. *J. Chem. Phys.* **107**, 8554–8560 (1997).

[52] Wilson, P. J., Bradley, T. J. & Tozer, D. J. Hybrid exchange-correlation functional determined from thermochemical data and ab initio potentials. *J. Chem. Phys.* **115**, 9233–9242 (2001).

[53] Toulouse, J. & Adamo, C. A new hybrid functional including a meta-gga approach. *Chem. Phys. Lett.* **362**, 72–78 (2002).

[54] Adamo, C. & Barone, V. Toward reliable adiabatic connection models free from adjustable parameters. *Chem. Phys. Lett.* **274**, 242–250 (1997).

[55] Adamo, C. & Barone, V. Exchange functionals with improved long-range behavior and adiabatic connection methods without adjustable parameters: The mPW and mPW1PW models. *J. Chem. Phys.* **108**, 664–675 (1998).

[56] Lynch, B. J., Fast, P. L., Harris, M. & Truhlar, D. G. Adiabatic connection for kinetics. *J. Phys. Chem. A* **104**, 4811–4815 (2000).

[57] Boese, A. D. & Handy, N. C. New exchange-correlation density functionals: The role of the kinetic-energy density. *J. Chem. Phys.* **116**, 9559–9569 (2002).

[58] Xu, X. & Goddard, W. A. The x3lyp extended density functional for accurate descriptions of nonbond interactions, spin states, and thermochemical properties. *Proc. Natl. Acad. Sci. U. S. A.* **101**, 2673–2677 (2004).

[59] Swart, M., Ehlers, A. W. & Lammertsma, K. Performance of the opbe exchange-correlation functional. *Mol. Phys.* **102**, 2467–2474 (2004).

[60] Zhao, Y., Schultz, N. E. & Truhlar, D. G. Exchange-correlation functional with broad accuracy for metallic and nonmetallic compounds, kinetics, and noncovalent interactions. *J. Chem. Phys.* **123**, 161103 (2005).

[61] Zhao, Y., Schultz, N. E. & Truhlar, D. G. Design of density functionals by combining the method of constraint satisfaction with parametrization for thermochemistry, thermochemical kinetics, and noncovalent interactions. *J. Chem. Theory Comput.* **2**, 364–382 (2006).

[62] Zhao, Y. & Truhlar, D. G. The m06 suite of density functionals for main group thermochemistry, thermochemical kinetics, noncovalent interactions, excited states, and transition elements: two new functionals and systematic testing of four m06-class functionals and 12 other functionals. *Theor. Chem. Acc.* **120**, 215–241 (2008).

[63] Zhao, Y. & Truhlar, D. G. The m06 suite of density functionals for main group thermochemistry, thermochemical kinetics, noncovalent interactions, excited states, and transition elements: two new functionals and systematic testing of four m06-class functionals and 12 other functionals. *Theor. Chem. Acc.* **120**, 215–241 (2008).
